# Hair Product Allergy: A Review of Epidemiology and Management

**DOI:** 10.7759/cureus.58054

**Published:** 2024-04-11

**Authors:** Abdullah N Alajaji

**Affiliations:** 1 Department of Dermatology, College of Medicine, Qassim University, Buraydah, SAU

**Keywords:** ammonium persulfate, para-phenylenediamine, occupational allergy, hair dye allergy, allergic contact dermatitis

## Abstract

Allergy to hair products is an increasingly common issue among people given the exposure to these products on a daily basis. Allergic reactions could be in the form of delayed-type contact dermatitis or the form of immediate-type hypersensitivity reactions. Hair products contain many ingredients and chemicals that patients may have allergies to, but common allergens are hair dyes, fragrances, persulfate salts, ammonium thioglycolate, coconut fatty acid derivatives, and acrylates. Allergy to hair dye is the most common followed by other allergens such as fragrances and persulfate salts. We discussed testing for hair dye allergy along with suggestions for alternative hair dyes that patients may use. Allergy to topical scalp medications is also seen in patients using those products. Allergy to topical minoxidil is seen more often due to the increased use of minoxidil sprays and foams among patients to increase hair growth. We will discuss in this review the diagnosis and alternatives for patients with minoxidil allergy. Hairdressers are at higher risk of allergy to hair products compared to the general population due to prolonged exposure to allergens and specific measures should be implemented to minimize the hazards of exposure.

## Introduction and background

Hair products are common causes of allergic reactions. Allergic contact dermatitis is the main type of allergic reaction caused by hair products. According to North American Contact Dermatitis Group (NACDG) data, allergies due to hair care products represented 9% of all allergies in patients who underwent patch testing [1]. The most common allergens in hair products are hair dyes followed by fragrance chemicals in shampoos and hair conditioners [1].

Given the wide variety of hair products that people use, there is an increasing need for a better understanding of these allergic reactions related to hair products with regard to types, major allergens, and alternatives for patients with allergies to these hair products. The scalp and neck are the most commonly involved areas in patients with contact dermatitis due to hair products. In hairdressers, hands are most commonly involved [2]. Delayed-type hypersensitivity in the form of contact dermatitis is the most common type of allergic reaction but immediate-type allergic reactions including anaphylaxis and contact urticaria are also seen in patients allergic to hair products, especially among hairdressers given long-term exposure to allergens via contact or inhalation type of exposure [3,4]. Standard patch test series include only a limited number of allergens in hair products and many positive allergic reactions are only seen after using extended patch testing that includes more hair product allergens [1].

In this review article, we will discuss the most common allergens in hair products and we will review the epidemiology and management of allergic reactions caused by hair products with a focus on hairdressers as a special population.

## Review

Epidemiology of hair product allergy

According to the North American Contact Dermatitis Group (NACDG) patch test study, allergic contact dermatitis from hair products represented 9% of all positive allergic reactions seen in 38770 patients tested between 2001-2016 [1]. Para-phenylenediamine (PPD) is the most frequent allergen in hair products followed by allergens in shampoos and conditioners [5]. Similar results were seen in other epidemiological studies done in other parts of the world such as Asia and Europe [6,7]. 

Based on NACDG data, 18% of patients, who had allergic contact dermatitis to hair products, had positive reactions to allergens not found in standard series thus implicating the importance of extended testing for patients with suspected allergy to hair products [1].

Allergens in hair dyes

Among all hair products, hair dye is the most common allergen to cause allergic contact dermatitis [5]. It is commonly used for social and cosmetic reasons. In the US and Europe, it is estimated that 50-80% of women and 10% of men over 40 years of age use hair dyes [8]. PPD is found in most hair dyes as it lasts longer giving better penetration to the hair cortex, but this deep penetration makes allergenicity of PPD higher [9]. The prevalence of PPD allergy among the general population is variable and ranges between 0.3% and 1.5% [1,10,11]. 

In Europe, 6% of the general population reports avoiding hair dyes due to skin problems related to hair dyes [11].

The prevalence of PPD-positive allergic reactions among dermatitis patients who underwent patch testing was found to be 6.2% in North America, 4% in Europe, and 4.3% in Asia [10,12,13]. According to NACDG patch test data, 4908 patients out of 38,775 patients tested, had allergic reactions to hair product allergens with PPD-positive reactions representing 36% of allergic reactions to hair products [10].

The most common presenting symptom of PPD hair dye allergy is scalp pruritus followed by an eczematous rash on the scalp, forehead, eyelids, and neck especially the nape area [14]. Patients may also have immediate-type allergic reactions that can present with urticarial, angioedema, or anaphylaxis [15,16].

Para-toluenediamine (PTD)-containing hair dyes can be used as an alternative to PPD hair dyes. PTD can cross-react with PPD causing allergic reactions in some patients but was tolerated by most [17,18].

Patients with positive allergic reactions to PPD are at higher risk of allergic reactions to other dyes. Park et al. showed that 55% of patients with an allergy to PPD tested positive for other allergens in the hair series compared to only 11% in the control group who did not have an allergy to PPD [19].

Kock et al. demonstrated that some patients with allergy to PPD/PTD may still be able to use the less sensitizing derivative of PPD called 2-methoxymethyl (ME)-PPD. They used 2-methoxymethyl-PPD in 43 patients with allergy to PPD/PTD and it was tolerated by 29 (67%) of them throughout continued hair dyeing with an average of nine treatments per year [20]. 

Patients with an allergy to PDD may use alternative dyes after doing a self-spot test to check for allergy prior to using it. Spot test can be done by applying a small amount of the hair dye product on a small (quarter size) area of skin preferably on the volar aspect of the forearm. After 45 minutes of contact, the product can be rinsed off. Skin should be checked for allergic reactions (redness or skin bumps) 15-20 minutes after washing the product off, and at 48-hour intervals for up to 4 to 6 days after application [21]. 

The gold standard for diagnosis of allergic contact dermatitis is via skin allergy patch testing. PPD is included in the standard T.R.U.E. patch test series. Alternative hair dyes that do not contain PPD can be used. Examples of PPD-free hair dye include Elumen by Goldwell and Palette by Nature [22]. Scheman et al. examined patients with PPD allergy to see if they tolerate PPD-free hair dyes that contain PTD and found that 57% of patients allergic to PPD were able to tolerate other hair dyes containing PTD [18]. Tammaro et al. examined the use of 2-methoxy methyl-PPD (Me-PPD), a chemical derivative of PPD in patients with documented allergy to PPD and showed that Me-PPD may be a safe alternative for patients with PPD allergy [23]. 

Permanent hair dyes utilize oxidative chemistry requiring the initiation of a chemical reaction by mixing chemicals just prior to application. These dyes typically contain 6%-9% hydrogen peroxide and ammonia as an alkalizing agent to increase the pH of the final product. This allows the hair dye to penetrate the cuticle and cortex and this can produce hair shaft damage and scalp irritation given deeper penetration ability [24]. 

Draelos et al. studied the use of an alternative monoethanolamine-based ammonia-free permanent cream hair dye without PPD in 50 female subjects using PTD as the primary intermediate and concluded that it is a safe alternative and resulted in less hair damage and less allergic reactions [24]. Symptoms of allergic contact dermatitis to PPD usually appear 1-3 days after contact in previously sensitized patients and at 4-14 days in patients with first exposure [25,26]. 

Immediate-type hypersensitivity reactions in the form of contact urticaria, asthma and even contact anaphylaxis due to hair dye have been reported [27]. Belton et al. reported a patient who had fatal anaphylaxis after exposure to PPD-containing hair dye. She had a history of anaphylaxis to a hair dye two years prior to her death [28]. 

In hairdressers, occupational hand dermatitis due to hair dyes can be managed by improving education, nitrile glove use, the application of after-work moisturizing creams, and topical steroids if needed [25]. 

Allergens in hair bleaching

This is a process of changing scalp hair color or getting hair highlighted in streaks. The first step of this process includes stripping hair of all eumelanin by mixing hydrogen peroxide, ammonia, and ammonium persulfate. The hair is then washed with a specialized shampoo. The next step is the application of hair color that may be a semi-permanent or a permanent dye [29]. 

Ammonium persulfate (APS) is an oxidizing agent used for multiple purposes including bleaching hair and bleaching flour. In 2020, We reviewed the prevalence of ammonium persulfate allergy among 2138 patients who underwent patch testing at Brigham and Women’s Hospital in Boston, and we found that 2.85% of our study sample had positive allergic reactions to APS [30]. According to the North American Contact Dermatitis Group patch test study that included 10526 between 2015 to 2018, allergy to APS was found to be 1.8% among patch-tested patients [31].

Ammonium persulfate is an important contact allergen and is associated with a higher prevalence of immediate hypersensitivity symptoms compared with other hair product allergens [32]. These symptoms include contact urticaria, rhinitis, bronchial asthma, and anaphylactic reactions. Piapan et al. reported that 13.6% of hairdressers were sensitized to ammonium persulfate [33]. Symanzik et al. reviewed bleaching powders and creams available in Germany and found that 16 out of 17 bleaching powders contained persulfates while 2 out of 6 bleaching creams contained persulfates [34]. 

According to a Finnish study that reviewed 290 hairdressers with occupational dermatitis, 15 patients had occupational contact urticaria, persulfate salts were the causative agents in 11 of these patients [35]. 

Due to persulfate exposure via inhalation, bleaching powders containing persulfates are the most common cause of occupational respiratory symptoms in hairdressers. These symptoms can be caused by an immediate hypersensitivity reaction or via non-immunologic irritation of the airways. Hairdressers have a 4-fold increased risk for wheezing and breathlessness than in matched controls from exposure to bleaching powder [36]. 

Lysdal et al. surveyed 5324 hairdressers in Denmark and showed that exposure to bleaching products was the most important factor for leaving the hairdressing profession due to associated respiratory symptoms [37]. 

 Özkaya et al. reviewed 55 hairdressers with occupational allergic contact dermatitis who were patch-tested in Turkey and found that 49% of them were allergic to ammonium persulfate [38]. 

Prevention of persulfate salts-induced allergy is via the use of safe bleach formulations such as persulfate free bleaching creams.

Hair straightening products

Ammonium thioglycolate is the main chemical used in straightening/waving hair products. It works on the disulfide bonds of hair to change hair structure. It has been used for many years and it is considered a rare allergen. Uter et al. showed in a study that allergy to ammonium thioglycolate was seen in 9 out of 702 hairdressers and in 6 of 1676 hair salon clients [39]. Patients with an allergy to ammonium thioglycolate-based hair-waving products may use alternative products that do not contain ammonium thioglycolate such as thiolactic acid-based products. 

Shampoos and conditioners

There are many causative ingredients in shampoos and conditioners that can cause allergic contact dermatitis. Fragrance is the main culprit but other allergens such as methylisothiazolinone (MI), methylchloroisothiazolinone (MCI), coconut fatty acid derivatives, propylene glycol, diazolinidyl urea, and formaldehyde are also causative agents. In shampoos, emulsifiers such as cetyl alcohol and lanolin alcohol as well as preservatives such as sodium benzoate, and benzalkonium chloride are potential allergens. Other common ingredients in shampoos/conditioners such as acrylates and antioxidants can be the culprit in hair product allergy [40]. 

The vast majority of shampoos and conditioners contain fragrance chemicals and the chemicals are common allergens in patients with scalp allergic contact dermatitis [5,41]. Certain shampoos are used commonly by skin of color individuals as they are created with ingredients that provide intense hydration, and protection for hair and scalp. Tawfik et al. reviewed allergens in 36 ethnic hair shampoos and 150 nonethnic shampoos and found that fragrance was present in 97% of these shampoos and reviewed 32 ethnic hair conditioners and 142 nonethnic conditioners, and fragrance was present in 96% and 98% of these conditioners, respectively [42]. Allergy to fragrances can be identified via baseline screening patch tests which include Fragrance Mix I (FM I) and Fragrance Mix II (FM II) along with balsam of Peru which will show fragrance chemicals that are most commonly involved in fragrance allergy. Other remaining fragrance chemicals could be identified via an extended fragrance series patch test. 

Surfactants are commonly present in hair products. Cocamidopropyl betaine (CAPB) and other related coconut fatty acid oil derivatives such as dimethylaminopropylamine (DMAPA) and cocamidopropyl dimethylamine are commonly used as surfactants in rinse-off cosmetic products such as shampoos and liquid soaps. In 2019, Weinhammer et al. reviewed 258 shampoos in the US market and CAPB was found in 162 (63%) of these shampoos [43]. 

Coconut derivatives most commonly cause irritant reactions and rarely cause a true allergic reaction. Suuronen et al. tested 1092 patients using coconut derivatives haptens and found that 39% of those patients had at least one irritant reaction while allergic reactions were seen in only 1.3% [44]. 

Of coconut derivatives, DMAPA is the most common culprit to cause allergic reactions. Foti et al. studied allergies related to coconut oil derivatives and found that DMAPA is the primary allergen [45]. Pham et al. reviewed 3185 patients who had scalp allergic contact dermatitis and found that cocamidopropyl betaine and 3-dimethylaminopropylamine represented 7% of those allergens [5]. 

CAPB is one of the coconut derivatives that can be the causative allergen in patients with allergy to shampoos. The reported prevalence of CAPB allergy in patch-tested patients ranges from 1.4% to 7.2% depending on the population studied [46,47]. Allergic patients may use alternative shampoos and conditioners that do not contain these coconut fatty acid derivatives.

Hair removal creams

Nanyan et al. reviewed fragrance allergens in 662 hair removal products available in the French market and found that fragrance allergens were present in 318 (48%) of these products with strip and spray formulations having significantly more allergens than cream products [48]. 

Hair extensions

Acrylates are present in cosmetic glues used in hair extensions, nail treatments, and eyelash extensions. The main acrylates used for these purposes are 2-hydroxyethyl methacrylate (HEMA) and ethyl cyanoacrylate. According to the North American Contact Dermatitis Group patch testing analysis report, 7% of 175 people with contact dermatitis due to nail care products tested positive for ethyl cyanoacrylate [49]. Uter et al. reviewed patch test results of 87 hairdressers and beauticians who perform hair extensions, nail treatments, and eyelash extensions and found that 27(31%) out of these 87 hairdressers had positive allergic reaction to 2-hydroxyethyl methacrylate [50]. Wetter et al. reviewed the patch test results of 871 patients tested for ethyl cyanoacrylate (ECA) and found that 1.1% of these patients showed positive allergic reaction to ECA [51]. Given increased exposure to acrylates among hairdressers, they are at higher risk of allergy as they use glue-containing acrylates in hair extensions. It is recommended that patients with an allergy to acrylate to use gloves made of neoprene or nitrile with neoprene as acrylic monomers can easily penetrate rubber (including latex) gloves and they only provide limited protection for a few hours given the penetration of acrylic monomers. Double gloving is recommended to increase protection [52].

Topical scalp medications

The main topical scalp medications that cause allergic reactions are topical minoxidil products due to long exposure and the presence of allergenic inactive ingredients. Topical minoxidil has been used for androgenic alopecia for many years and it was shown to be effective. Common side effects of topical minoxidil products include dryness, scaling of the scalp, and itching. Allergic contact dermatitis to topical minoxidil products can be due to the active ingredient or inactive ingredients such as propylene glycol. Allergic contact dermatitis to topical minoxidil products is mostly due to the inactive ingredient propylene glycol but there are several reports of allergic contact dermatitis caused by the active ingredient of topical minoxidil including a case of pustular allergic reaction that was proven by patch test [53]. For patients with suspected allergy to topical minoxidil products, patch testing to both active ingredients (minoxidil) and inactive ingredient (propylene glycol) is recommended. If the allergy was due to propylene glycol, the patient may use an alternative minoxidil formulation without propylene glycol [54].

Oral minoxidil could be used as an alternative for patients with allergy to topical minoxidil. Therianou et al. recently reviewed nine patients with proven allergies to the active ingredient of minoxidil and a negative patch test to propylene glycol. The authors studied the safety of using oral minoxidil in these nine patients who had documented allergy to topical minoxidil and after an average use of 17 months, none of these patients showed allergic reactions [55].

Hairdressers as a special population exposed to allergens

Due to prolonged exposure to allergens in hair products, hairdressers are at a much higher risk of allergy compared to the general population. Different allergens are associated with different hair procedures such as hair washing, styling, waving, dying, and bleaching hair.

Kieć-Swierczyńska et al. reviewed patch test results of 121 hairdressers and found that 69% had at least 1 positive allergic reaction with 25% of those positive reactions attributed to p-phenylenediamine, 23% due to ammonium persulfate, 9.8% to 2,5-diaminotoluene sulfate and 7% to ammonium thioglycolate [56]. Jamil et al. studied the incidence of hand eczema in different occupations and found that hairdressers ranked first among other occupations in the incidence of occupational contact dermatitis followed by nurses and metal workers [57].

Contact dermatitis in hairdressers can be managed by improving education regarding allergens, the application of after-work moisturizing creams, and the use of nitrile gloves unless they are allergic to acrylates as monomers can penetrate gloves [57].

Allergic reactions commonly result in delayed-type hypersensitivity reactions causing allergic contact dermatitis, but immediate-type hypersensitivity can occur especially with exposure to persulfate salts during hair bleaching. Immediate type hypersensitivity could be in the form of contact urticaria, respiratory symptoms, or even anaphylactic reactions [58]. Moscato, et al. studied 47 hairdressers over a period of eight years and found that 24 out of 47 received a diagnosis of occupational asthma which was due to persulfate salts in 87% of them and to permanent hair dyes in 8% [59]. Hair dyes can also cause immediate-type hypersensitivity reactions but are less common than persulfate salts. Helaskoski et al. reviewed patient files of Finnish Institute of Occupational Health to identify patients diagnosed with immediate-type hypersensitivity due to hair dyes and found 11 hairdressers with five of them having occupational asthma, five with rhinitis, and three with contact urticaria [60].

Hairdressers are exposed to acrylates during hair extension procedures and have a 9-fold increased risk of developing contact allergy to 2-hydroxyethyl methacrylate compared to the general population [61].

Franic et al. studied the prevalence of occupational contact dermatitis among 408 hairdressing apprentices during schooling and found that 15% of all tested students had an allergy to PPD, 11% to ammonium persulfate and 4% to both toluene-2,5-diamine sulfate and ammonium thioglycolate [62].

Management of allergy to hair products

Patients with allergic contact dermatitis to hair products most commonly present with itchy, eczematous rash generally confined to the site of contact with the allergen. Patch testing is the gold standard for diagnosing allergic contact dermatitis. Screening patch tests include the standard T.R.U.E. patch test and North American 80 comprehensive patch test which is more sensitive as it contains more haptens. An extended patch test series can be ordered according to the patient's complaint and susbected allergens. The first read of the patch test is usually done at 48 hours and the final read can be done at 72 hours or 96 hours. If patients are symptomatic and while waiting for patch test, they should be advised to avoid applying topical hair care products on affected areas until the causative chemicals are identified via patch test. Meanwhile, a topical steroid can be used to treat affected areas. 

If the patient is suspicious about a particular hair care product as the cause of contact dermatitis, a repeat open application test (ROAT) can be done. It has a similar principle to patch tests and can be done to test cosmetic and hair products. Patients can apply the product to a small hairless skin area such as the side of the forearm or behind the ear. The product is left open, and in the case of shampoos and cleansers, the product should be washed off after 1-2 minutes. The product is reapplied to the same area twice a day for 1 week. At the end of the week, if the patient is allergic to that product, the itchy rash will appear at the site of application [63].

PPD is the most common allergen in hair products. Treatment of PPD allergy is dependent on presenting symptoms. If itching and eczematous rash are present, patients can be treated with topical steroids and they need to avoid using PPD hair dye. An alternative PPD-free hair dye can be used after doing a self-test of the new hair dye. Examples of alternative (PPD free) hair dyes are listed in Table 1.

**Table 1:** Recommendation to minimize risk of contact dermatitis in hairdressers PPD: Para-phenylenediamine

Steps	Measures		
1.	Substitute allergens with safe alternatives	Allergens	Alternatives
		PPD containing hair dye	Use PPD-free alternative hair dye such as monoethanolamine based hair dye, Para-toluenediamine (PTD) containing hair dye or 2-methoxymethyl (ME)-PPD hair dye
		Persulfates in bleaching powders	Use persulfate-free bleaching creams
		Ammonium thioglycolate in hair perm products	Use cysteamine containing hair perm products
2.	Engineering measures to limit exposure such as ensuring a well-ventilated room for mixing hair products.		
3.	Administrative measures and policies to minimize exposure to allergens such as distributing the wet work evenly among employees to reduce the individual’s exposure to wet work.		
4.	Use of personal protective equipment such as wearing gloves at work.		

Washing hair with the head held back would decrease the exposure to scalp products such as hair dye hereby minimizing the risk of allergy.

Fragrance chemicals are present in almost all shampoos and conditioners and in most other hair products, finding fragrance-free products can be difficult. There are websites that can help patients search for alternative shampoos/conditioners without fragrance chemicals. Examples of these websites include the Contact Allergen Management Program (CAMP) developed by the American Contact Dermatitis Society and the Contact Allergen Replacement Database (CARD) developed by the Mayo Clinic. These websites are helpful as they provide patients with available alternative products that do not contain their allergens. In the case of fragrance allergy, one of the options that do not contain fragrance is Free and Clear shampoo by Vanicream.

Coconut oil derivatives such as DMAPA and CAPB rarely cause allergic reactions as they mostly cause irritant reactions. Almost all shampoos contain coconut derivatives as they function as surfactants for cleansing and foam boosters in shampoos. In the rare situation of a true allergy, patients may use a cleanser that does not contain coconut derivatives such as skin calming body wash by Eucerin which can be used on the scalp for patients with a true allergy to coconut derivatives. 

Localized scalp contact dermatitis can be treated with topical steroids. Topical tacrolimus can be used as an alternative treatment in patients with an allergy to topical steroids [64].

Patients with extensive dermatitis and severe symptoms can be treated with a short course of oral prednisone for a few days. Chronic contact dermatitis is usually treated with intermittent topical steroids or topical tacrolimus but if symptoms persist narrowband ultraviolet B phototherapy can be used [65].

Recommendations to minimize the risk of contact dermatitis in hairdressers along with suggested alternatives to allergens in hair products are summarized in Table 1.

## Conclusions

Hair products contain many allergens that can potentially cause contact dermatitis as well as immediate-type hypersensitivity reactions. PPD, fragrance, and ammonium persulfates are the most common allergens found in hair products. The gold standard test to diagnose allergic contact dermatitis is via patch test. Most people use hair products on a daily basis; therefore, it is important to identify and avoid culprit allergens in hair products and use alternative products instead. Hairdressers are at a much higher risk of hair product allergy due to their continuous exposure. Measures should be implemented to minimize the hazards of exposure to these hair products.
